# Numerical Study of the Effects of Residual Stress on Fretting Fatigue Using XFEM

**DOI:** 10.3390/ma8105365

**Published:** 2015-10-19

**Authors:** Huayang Zhang, Jinxiang Liu, Zhengxing Zuo

**Affiliations:** School of Mechanical Engineering, Beijing Institute of Technology, Beijing 100081, China; huayangzhang2009@gmail.com (H.Z.); zxzuo@bit.edu.cn (Z.Z.)

**Keywords:** residual stress, fretting fatigue, extended finite element method, cyclic cohesive zone model

## Abstract

Residual compressive stress can improve fretting fatigue strength. In this paper, the effects of residual stress on fretting fatigue of Al 2024-T351 alloy specimens are studied using a numerical approach. The extended finite element method combined with the cyclic cohesive zone model is adopted to model fretting fatigue crack growth behavior. It is shown that residual stress changes the fretting fatigue crack growth path and enhances fretting fatigue life. Crack initiation angle, depth of knee point, crack initiation life, crack propagation life and total life are greater for specimens with residual stress compared to specimens without residual stress. The effects of residual stress are more remarkable for specimens with a high intensity of residual stress. However, the effects of residual stress reduce at a high bulk load level.

## 1. Introduction

Fretting fatigue results from small amplitude oscillatory movement between contacting bodies. Due to stress concentration, the life of components under the fretting fatigue condition is lower than that under the plain fatigue condition [1]. Many crucial components, such as bolted joints and blade-disk dovetail connections in gas turbine engines, fail owing to fretting fatigue [2,3,4]. Several methods, such as laser shock peening, shot peening and deep rolling, can not only enhance plain fatigue strength [5], but also improve fretting fatigue strength [6,7,8,9]. These methods induce residual compressive stress in the material surface. The residual compressive stress is capable of delaying or even diminishing fatigue crack initiation and growth, and the fretting fatigue life of components is therefore enhanced.

Some studies have been carried out on the fretting fatigue of specimens with residual stress experimentally. Experimental methods can explicitly provide crack initiation locations. According to the examination of fracture surfaces with scanning electron microscopy, the crack initiation occurs at the contact surface for specimens without residual stress or with a low intensity of residual stress. For specimens with a high intensity of residual stress, it is also possibly for cracks to initiate inside the specimen [10]. However, considering that crack initiation locations are usually covered by pads, it is difficult to estimate the precise crack initiation moment. Detailed information about crack propagation is also barely given by means of experimental examination. In addition, for experimental systems, some inherent defects cannot be overcome, and therefore, it is difficult to meet the requirements occasionally. For example, there is a limit to the stiffness that can be designed into the beams constituting springs for a one-actuator hydraulic testing machine [11].

Based on experimental results, numerical techniques are capable of dealing with some problems that cannot be overcome in experiments to some extent. The finite element method (FEM) is a very popular method, which has been widely used in many fields [12,13,14]. Several researchers investigated fretting fatigue crack propagation behavior by utilizing FEM [15,16]. It is shown that residual stress can improve fretting fatigue life significantly. FEM can be used to monitor the crack propagation behavior of specimens incrementally. However, some problems are inevitable in FEM. First, when the crack path is unknown or the mode mixity changes, FEM is powerless to model crack growth. Second, remeshing is essential with crack growth in order to form the new crack tip. Due to stress singularity in the crack tip, a very fine mesh is used near the crack tip. As a result, the computing cost is excessive.

Recently, the extended finite element method (XFEM), which is based on the partition of unity, has been introduced [17,18]. In the frame of XFEM, the crack growth path is independent of mesh geometry, and remeshing is not needed. Therefore, XFEM can be used to model a crack for which the path is unknown and for which the mode mixity is variational, and the computing cost is reduced. However, stress singularity in the crack tip still exists. Stress singularity can be avoided in the cohesive zone model. The fundamental idea of the cohesive zone model can be traced to the strip yield models of Dugdale and Barenblatt [19,20]. There is a fracture process zone in front of the crack tip during crack growth. The material response in the aforementioned zone is different from that in the bulk material. In this zone, material obviously degrades, and the traditional constitutive equation in bulk material is unsuitable. The cohesive zone model is capable of characterizing material degradation in the fracture zone in front of the crack tip.

This paper adopts XFEM combined with the cyclic cohesive zone model (CCZM) to study the effects of residual stress on fretting fatigue and is organized as follows. First, XFEM and CCZM are introduced, respectively. Next, the numerical modeling of fretting fatigue is detailed. Then, the effects of residual stress on fretting fatigue crack initiation angle, depth of knee point, crack initiation life, crack propagation life and total life are described. Finally, some conclusions are provided.

## 2. Theory Background

### 2.1. Extended Finite Element Method

**Figure 1:**
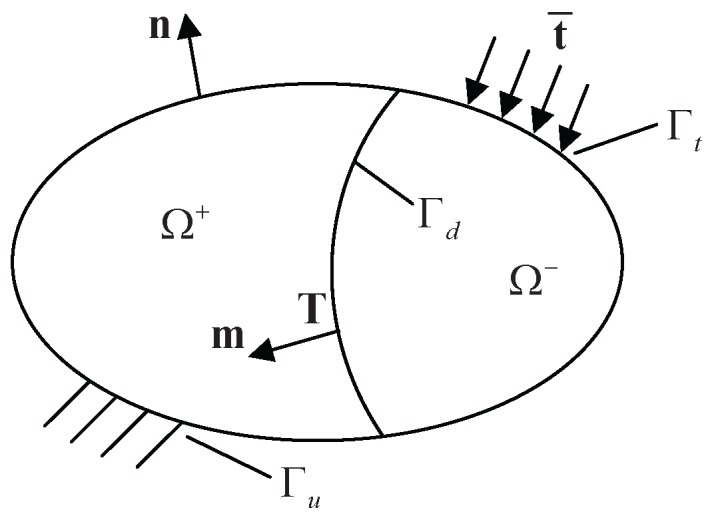
Domain Ω crossed by a discontinuity Γ_d_ and corresponding boundary conditions.

Take into account a domain Ω, which is divided into two parts (Ω^+^ and Ω^−^) by a discontinuity Γ_d_, as shown in Figure 1. The displacement field **u** is composed of the continuous and discontinuous parts by:

(1) u=Na+HNb

where **N** is a matrix containing the conventional shape functions. **a** denotes the regular displacement nodal degrees of freedom (DOFs) and **b** represents the enriched nodal DOFs for the displacement field. *H* is a Heaviside function and is defined as:

(2) $$H=\left\{\begin{array}{c} 1\;\;\;\;\;\;\;\;\text{for}\;\;\;\;x\in \Omega^{+}\\ 0\;\;\;\;\;\;\;\;\text{for}\;\;\;\;x\in \Omega^{-} \end{array}\right.\;$$

Boundary conditions are written as:

(3) $$\begin{array}{c} \sigma \cdot n=\bar{t}\;\;\;\;\;\;\;\;\;\;\;\;\;\;\;\text{on}\;\;\;\;\Gamma_{t}\\ \sigma \cdot m=T\;\;\;\;\;\;\;\;\;\;\;\;\text{on}\;\;\;\;\Gamma_{d}\\ u=\bar{u}\;\;\;\;\;\;\;\;\;\;\;\;\;\;\;\;\;\;\;\;\;\;\;\;\;\;\;\text{on}\;\;\;\;\Gamma_{u} \end{array}\;$$

where *σ* represents the Cauchy stress tensor. **n** is the outward unit normal vector of the boundary. $\bar{t}$ denotes the external traction at the boundary Γ_t_. **m** is the inward unit vector, which is normal to the discontinuity Γ_d_. **T** stands for the internal traction at Γ_d_. $\bar{u}$ is the prescribed displacement and applied on the boundary Γ_u_.

The XFEM equation can be derived from the principle of virtual work, *i.e.*,

(4) $$\delta \Pi_{i}=\delta \Pi_{e}$$

where *δ* represents the variation of a quantity. $\delta \Pi_{i}$ and $\delta \Pi_{e}$ are the internal virtual work and the external virtual work, respectively.

Combined with the above equations, the final XFEM equation is expressed as [21]:

(5) $$\left[\begin{array}{cc} K_{\mathrm{aa}} & K_{\mathrm{ab}}\\ K_{\mathrm{ba}} & K_{\mathrm{bb}} \end{array}\right]\left[\begin{array}{c} \Delta a\\ \Delta b \end{array}\right]=\left[\begin{array}{c} f_{\mathrm{ea}}\\ f_{\mathrm{eb}} \end{array}\right]-\left[\begin{array}{c} f_{\mathrm{ia}}\\ f_{\mathrm{ib}} \end{array}\right]\;$$

The corresponding terms are given by:

(6) $$\begin{array}{c} K_{\mathrm{aa}}=\int_{\Omega }B^{T}DBd\Omega \\ K_{\mathrm{ab}}=K_{\mathrm{ba}}=\int_{\Omega^{+}}B^{T}DBd\Omega \\ K_{\mathrm{bb}}=\int_{\Omega^{+}}B^{T}DBd\Omega +\int_{\Gamma_{d}}N^{T}\mathrm{CN}d\Gamma \\ f_{\mathrm{ea}}=\int_{\Gamma_{t}}N^{T}\bar{t}d\Gamma \\ f_{\mathrm{eb}}=\int_{\Gamma_{t}^{+}}N^{T}\bar{t}d\Gamma \\ f_{\mathrm{ia}}=\int_{\Omega }B^{T}\sigma d\Omega \\ f_{\mathrm{ib}}=\int_{\Omega^{+}}B^{T}\sigma d\Omega +\int_{\Gamma_{d}}N^{T}Td\Gamma \end{array}\;$$

where **C** is the cohesive stiffness matrix and expressed as:

(7) $$C=\left[\begin{array}{cc} \frac{\partial T_{n}}{\partial \delta_{n}} & \frac{\partial T_{n}}{\partial \delta_{t}}\\ \frac{\partial T_{t}}{\partial \delta_{n}} & \frac{\partial T_{t}}{\partial \delta_{t}} \end{array}\right]\;$$

where $T_{n}$ and $T_{t}$ are the normal component and the shear component of **T**, respectively. $\delta_{n}$ and $\delta_{t}$ are the normal separation and the shear separation, correspondingly. The cyclic cohesive zone model will be illustrated in detail in the next section. In addition, Δa and Δb are the increment of displacements. **D** and **B** are the material matrix and the strain matrix, respectively. Residual stress is embedded in *σ* of Equation (6) at the beginning of the whole computation.

### 2.2. Cyclic Cohesive Zone Model

The mixed mode is involved for fretting fatigue crack growth [22]. Thus, in order to reasonably describe the fretting fatigue crack, the force connecting cohesive crack surfaces should contain normal traction and shear traction simultaneously in CCZM. In the present study, a popular cohesive law is employed and expressed as [23]:

(8) $$\begin{array}{l} T_{n}=\sigma_{\max,0}\;e\;\exp\left(-\frac{\delta_{n}}{\delta_{0}}\right)\left\{\frac{\delta_{n}}{\delta_{0}}\;\exp\left(-\frac{\delta_{t}^{2}}{\delta_{0}^{2}}\right)+\left(1.0-q\right)\;\frac{\delta_{n}}{\delta_{0}}\;\left[1.0-\exp\left(-\frac{\delta_{t}^{2}}{\delta_{0}^{2}}\right)\right]\right\}\\ T_{t}=\tau_{\max,0}\;\sqrt{2e}\;\frac{\delta_{t}}{\delta_{0}}\;\left(1.0+\frac{\delta_{n}}{\delta_{0}}\right)\exp\left(-\frac{\delta_{n}}{\delta_{0}}\right)\exp\left(-\frac{\delta_{t}^{2}}{\delta_{0}^{2}}\right) \end{array}\;$$

where $T_{n}$ and $T_{t}$ are the normal traction and shear traction, respectively. $\delta_{n}$ and $\delta_{t}$ are the normal separation and shear separation, respectively. The initial normal cohesive strength, $\sigma_{\max,0}$, is the peak value of the normal traction under monotonic and pure normal loading conditions. Similarly, the initial shear cohesive strength, $\tau_{\max,0}$, represents the maximum value of the shear traction under monotonic and pure shear loading conditions. Here, $\tau_{\max,0}=\sqrt{2e}q\sigma_{\max,0}$. The cohesive length, $\delta_{0}$, denotes the separation where the normal traction reaches the cohesive strength in normal loading. *e* is the Eulerian number, *i.e.*, *e* = exp(1). The definition of the parameter *q* is provided subsequently.

Under monotonic and pure normal loading conditions, the normal traction $T_{n}$ in Equation (8) turns into:

(9) $$T_{n}=\sigma_{\max,0}\;e\left(\frac{\delta_{n}}{\delta_{0}}\right)\exp\left(-\frac{\delta_{n}}{\delta_{0}}\right)$$

Similarly, under monotonic and pure shear loading conditions, the shear traction $T_{t}$ in Equation (8) becomes:

(10) $$T_{t}=2\sigma_{\max,0}\;e\;q\left(\frac{\delta_{t}}{\delta_{0}}\right)\exp\left(-\frac{\delta_{t}^{2}}{\delta_{0}^{2}}\right)$$

Integrating Equations (9) and (10), the work of normal separation $\phi_{n,0}$ and the work of shear separation $\phi_{t,0}$ under monotonic loading conditions can be obtained, *i.e.*, $\phi_{n,0}=e\sigma_{\max,0}\delta_{0}$, $\phi_{t,0}=\sqrt{\frac{e}{2}}\tau_{\max,0}\delta_{0}$. The parameter *q* is defined as $q=\phi_{t,0}/\phi_{n,0}$.

In order to depict the degradation behavior of material under cyclic loading conditions, a damage variable, *D*, is introduced, and the incremental form ΔD is written as:

(11) $$\Delta D=\frac{\left|\Delta \delta \right|}{\delta_{\Sigma }}\left(\frac{T}{\sigma_{\max}}-\frac{\sigma_{f}}{\sigma_{\max,0}}\right)H\left(\delta_{c}-\delta_{0}\right)\;\;\;\;\;\;\;\;\mathrm{and}\;\;\;\;\Delta D>0$$

where Δδ is the separation increment. $\delta_{c}$ stands for accumulated separation. *T* denotes the resultant traction. *H* represents the Heaviside function. $\sigma_{f}$ and $\delta_{\Sigma }$ are the cohesive zone endurance limit and the accumulated cohesive length, respectively. The material is cracked when *D* reaches one. The current cohesive strengths depend on the current state of damage and are defined as:

(12) $$\begin{array}{c} \sigma_{\max}=\sigma_{\max,0}\left(1-D\right)\\ \tau_{\max}=\tau_{\max,0}\left(1-D\right) \end{array}\;$$

Under cyclic loading conditions, the initial cohesive strengths need to be replaced by the current cohesive strengths.

The definition of the unloading/reloading path is essential for CCZM. This path follows a linear relationship with a slope equal to that of the current traction separation curve at zero separation. The current unloading/reloading stiffness $k_{n}$ (normal) and $k_{t}$ (shear) are written as:

(13) $$\begin{array}{c} k_{n}=e\frac{\sigma_{\max}}{\delta_{0}}\\ k_{t}=\sqrt{2e}\frac{\tau_{\max}}{\delta_{0}} \end{array}\;$$

It is important to define the crack growth direction for fretting fatigue specimens. Considering the mode mixity for fretting fatigue crack, the range of shear stress and the range of normal stress seem to be more appropriate parameters for predicting the crack growth direction compared to others [24]. In this study, a two-stress criterion, which includes the range of shear stress and the range of normal stress, is used to judge the crack growth path. This criterion is expressed as:

(14) $$\alpha =\left\{\begin{array}{c} \alpha_{n},\;\mathrm{if}\;\;\;\frac{{\left(\Delta T_{n}\right)}_{\max}-\sigma_{e}}{\sigma_{e}}>\frac{{\left(\Delta \left|T_{t}\right|\right)}_{\max}-\tau_{e}}{\tau_{e}}\;\;\mathrm{and}\;\;T_{n}>\sigma_{e}\\ \alpha_{n}+\frac{\pi }{4},\;\mathrm{if}\;\;\;\frac{{\left(\Delta T_{n}\right)}_{\max}-\sigma_{e}}{\sigma_{e}}<\frac{{\left(\Delta \left|T_{t}\right|\right)}_{\max}-\tau_{e}}{\tau_{e}}\;\;\mathrm{and}\;\;\left|T_{t}\right|>\tau_{e} \end{array}\right.\;$$

where $\sigma_{e}$ and $\tau_{e}$ are the crack endurance limits of tensile and shear mechanisms, respectively. *α* is the fretting fatigue crack growth direction. $\alpha_{n}$ represents the angle when the range of shear stress $\Delta \left|T_{t}\right|$ reaches its minimum value ${\left(\Delta \left|T_{t}\right|\right)}_{\min}$ and the range of normal stress $\Delta T_{n}$ reaches its maximum value ${\left(\Delta T_{n}\right)}_{\max}$.

## 3. Numerical Modeling of Fretting Fatigue

**Figure 2:**
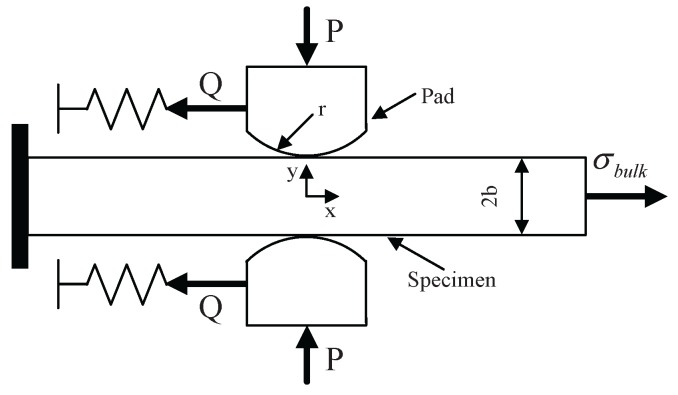
Schematic of the fretting fatigue model. P is normal load, Q is tangential load and *σ*_bulk_ is bulk load.

**Figure 3:**
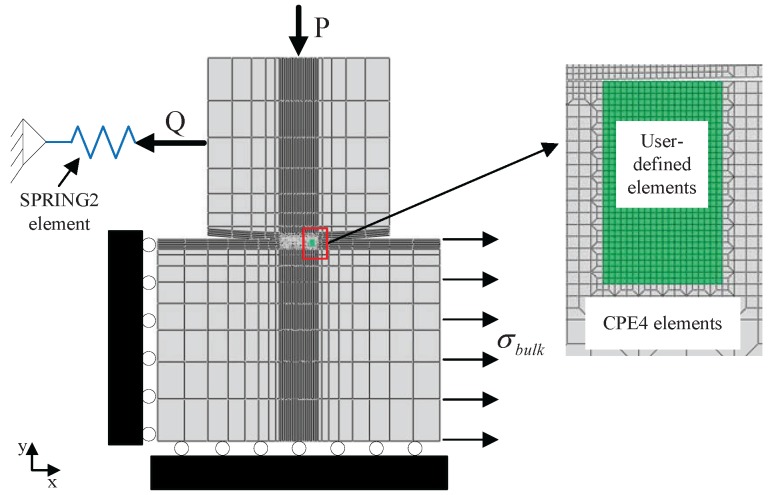
Fretting fatigue global model and the magnified figure of the user-defined elements.

This paper models one-actuator fretting fatigue, as shown in Figure 2. The cylindrical end radius of the pad is 50 mm. The specimen width *w* and thickness 2*b* are 10 and 20 mm, respectively. Considering the symmetry of the specimen and loading conditions, only one pad and half of the specimen are employed in the numerical model. The numerical model of fretting fatigue is displayed in Figure 3. XFEM and CCZM are embedded in the user-defined subroutine UEL of the finite element software ABAQUS (Dassault Systemes Simulia Corp., Providence, RI, USA) in order to model fretting fatigue crack growth. Six hundred eighty user-defined elements, which are controlled by the UEL subroutine, are used in the zone that the crack possibly crosses. The element size is 0.01 mm × 0.01 mm. The pad and other zones of the specimen adopt 2770 conventional four-node plain stain elements (CPE4). In addition, a spring element (SPRING2) is used.

The displacement boundary conditions are listed as follows: for the specimen, the displacement along the *x* direction of the left side and the displacement along the *y* direction of the bottom side are constrained. *x* and *y* are shown in Figure 3. For the pad, multi-point constraints (MPC) at the top node of the pad and the node of the spring are established in order to make sure that their displacement in the *y* direction is the same. In addition, MPC in the *x* direction is applied at the left side of the pad. For the spring, the displacement along the *x* direction of its left node is restricted. In one-actuator fretting fatigue tests, bulk load $\sigma_{\mathrm{bulk}}$ is controlled by the actuator. $\sigma_{\mathrm{bulk}}$ is imposed on the right side of the specimen, and tangential load *Q* is produced by the spring accordingly.

**Figure 4:**
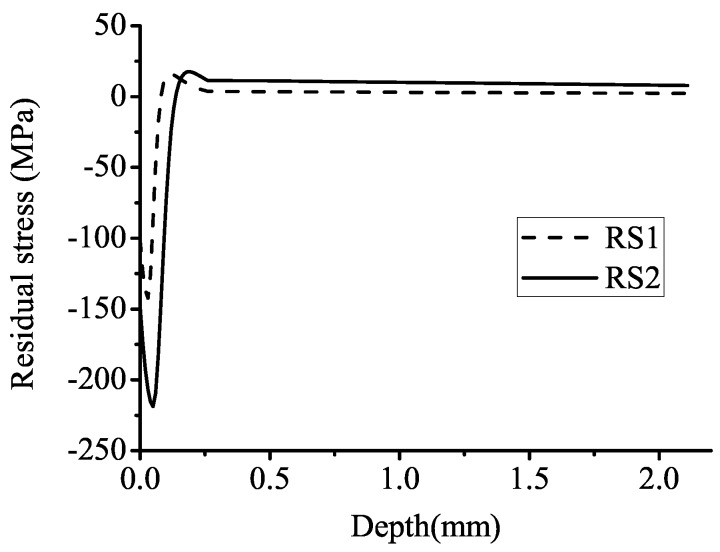
Residual stress profiles. RS1 and RS2 denote a low and a high intensity of residual stress, respectively.

In order to study the effects of residual stress and bulk load on fretting fatigue behavior, two different intensities of residual stress additional to the situation without residual stress and two different intensities of bulk load are chosen. Two groups of residual stress $\sigma_{\mathrm{RS}}$ are selected based on an interval function in [25]. Residual stress profiles are shown in Figure 4. RS1 and RS2 denote a low and a high intensity of residual stress, respectively. The residual stress at the specimen surface is −100 and −150 MPa, respectively. The maximum residual compressive stress ($\sigma_{\mathrm{MRCS}}$) is −140 and −220 MPa, respectively. Different bulk loads of 150 and 200 MPa are selected, and the stress ratio of both bulk loads is zero in all computational cases. A normal load *P* of 3.6 kN keeps constant in the loading steps. The coefficient of friction *μ* and the spring stiffness *K* are set to 0.75 and 560,060 N/mm, respectively. Here, *K* is calibrated to make sure that *Q*/μ*P* < 1.0, so that fretting behavior is under partial slip conditions in all loading situations. Computational cases are listed in Table 1.

In this paper, the specimen and pad are Al 2024-T351 alloy. The constitutive relation in the bulk material adopts the $J_{2}$ theory. The same elastic-plastic constitutive relation is used in user-defined elements (based on XFEM technology) and ABAQUS built-in elements. The mechanical properties of the Al 2024-T351 alloy are listed as follows: elastic modulus *E* = 72,400 MPa, Poisson’s ratio *ν* = 0.33, fatigue limit $\sigma_{d}$ = 140 MPa, yield strength $\sigma_{y}$ = 324 MPa and ultimate tensile strength $\sigma_{u}$ = 428 MPa [26,27].

**Table 1:** Details of the computational cases.

Cases	*σ*_MRCS_ (MPa)	*σ*_bulk_ (MPa)	*Q*/*μP*
T1	0	150	0.6
T2	−140	150	0.6019
T3	−220	150	0.6020
T4	0	200	0.7671
T5	−140	200	0.7732
T6	−220	200	0.7808

For the potential crack path, the constitutive relation employs CCZM. CCZM parameters are set as follows. For the specimen under the plane strain condition, the value of $\sigma_{\max,0}$ is usually located between three- and four-times the material yield strength $\sigma_{y}$ [28]. Here, $\sigma_{\max,0}$ is considered as 3$\sigma_{y}$, *i.e.*, $\sigma_{\max,0}$ = 972 MPa. The work of normal separation can be identified with the J-integral, *i.e.*, $\phi_{n,0}=J_{\mathrm{IC}}$. $J_{\mathrm{IC}}$ is given as:

(15) $$J_{\mathrm{IC}}=\frac{1-\nu^{2}}{E}K_{\mathrm{IC}}^{2}\;$$

Here, fracture toughness $K_{\mathrm{IC}}$ = 37 MPa $\sqrt{m}$ [26]. Thus, the cohesive length can be determined, and its value is $\delta_{0}$ = 0.006377 mm. The cohesive zone endurance limit and the accumulated cohesive length are assumed to be $\sigma_{f}=0.03\sigma_{\max,0}$ and $\delta_{\Sigma }=6\delta_{0}$, respectively.

## 4. Results and Discussion

### 4.1. Crack Growth Path

For specimens without residual stress (T1 and T4), the crack initiates near the trailing edge of contact (*x*/*a* = 1.0) due to a high stress concentration, which has been proven by many researchers [29,30]. Here, *a* is the contact half-width and equals 0.75 mm. For specimens with a low intensity of residual stress (T2 and T5), the crack also starts in the vicinity of the trailing edge of contact. For specimens with a high intensity of residual stress (T3 and T6), the crack not only initiates near the trailing edge of contact, but also emanates from inside the specimen, where the residual tensile stress reaches its maximum value. A similar crack initiation location has been observed in [31].

**Figure 5:**
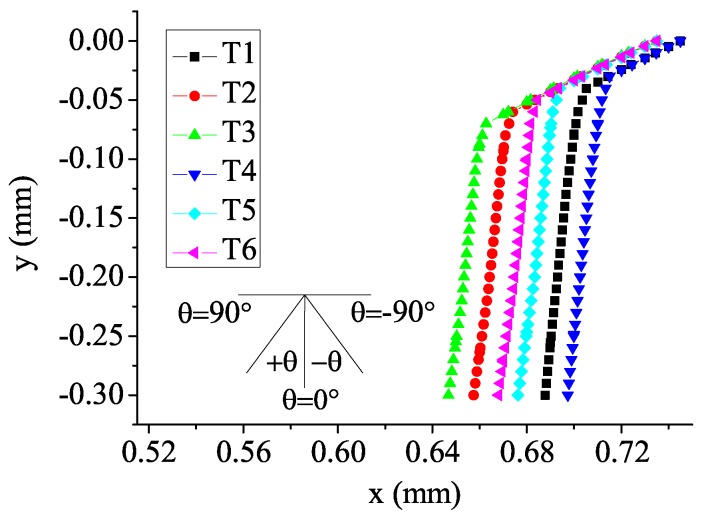
Schematic of crack initiation angle and growth path.

In the study, the potential crack path is estimated based on the stress state before crack growth. Subsequent computation is used to obtain the crack growth rate. The details of crack initiation angle and growth path are shown in Figure 5. In Figure 5, the horizontal axis and the vertical axis represent the *x* coordinate and the *y* coordinate, respectively. The origin of the coordinate is located in the center of the contact surface (see Figure 3). Each point in Figure 5 represents the coordinate of the intersection between the crack line and the initial square element edge. Values of crack initiation angles are listed in Table 2. Under the bulk load of 150 MPa condition, crack initiation angles in specimens with a low and a high intensity of residual stress (T2 and T3) are increased by 4.3% and 6.4% in comparison with specimens without residual stress (T1), respectively. Under the bulk load of 200 MPa condition, crack initiation angles in specimens with a low and a high intensity of residual stress (T5 and T6) are increased by 2.2% and 4.3% in comparison with specimens without residual stress (T4), respectively. Therefore, under the condition of the same bulk load, crack initiation angles are greater for specimens with residual stress compared to specimens without residual stress, and the effects of residual stress are more obvious for specimens with a high intensity of residual stress. This is because considering the residual compressive stress near the specimen surface, the tensile stress in the specimen is offset partially. Accordingly, the crack is prone to initiate in the angle that is close to the horizontal direction and away from the vertical direction. Waterhouse’s research confirms this trend [32]. In addition, for specimens with the same group of residual stress, crack initiation angles are greater at a low bulk load level. Therefore, the influences of residual stress on crack initiation angles decrease at a high bulk load level.

**Figure 6:**
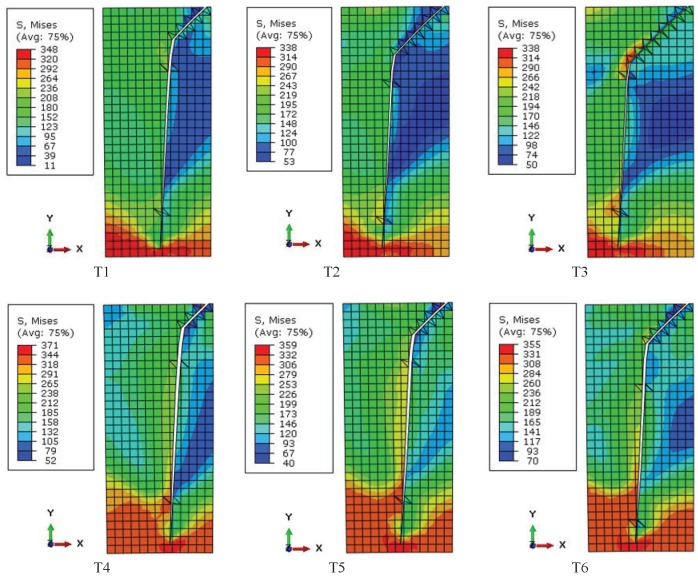
Fretting fatigue crack growth path in the numerical model (stress units: MPa).

Residual stress not only influences crack initiation angles, but also affects the crack growth path. During the early stage of crack growth, shear stress is responsible for crack growth. For specimens without residual stress (T1 and T4), the effects of bulk load intensify and the influences of contact load reduce with crack propagation, and the crack propagation orientation transforms from oblique to normal. Then, the normal crack, which is dominated by normal stress, advances towards the inside of the specimen. For specimens with residual stress (T2, T3, T5, T6), the depth of the knee point is increased in that the tensile stress is partially counteracted by residual compressive stress. As Figure 5 suggests, the depths of the knee point are 0.04, 0.06, 0.07, 0.03, 0.04, 0.05 mm from T1 to T6, respectively. Under the bulk load of 150 MPa condition, the depths of the knee point in specimens with a low and a high intensity of residual stress (T2 and T3) grow by 50% and 75% compared to the specimen without residual stress (T1), respectively. Under the bulk load of 200 MPa condition, the depths of the knee point in specimens with a low and a high intensity of residual stress (T5 and T6) grow by 33.3% and 66.7% compared to the specimen without residual stress (T4), respectively. In addition, for specimens with the same group of residual stress, the knee point is deeper at a low bulk load level. Accordingly, the effects of residual stress on the knee point depth are more obvious under the high intensity of residual stress condition and at a low bulk load level. The knee point position and the crack growth path after material failure in the numerical model are shown in Figure 6.

In addition, taking into account two initiation sites for specimens with a high intensity of residual stress (T3 and T6), the crack growth path is not along one direction. When the surface crack reaches the depth of 0.06 mm at a low bulk load level (T3) or 0.07 mm at a high bulk load level (T6), an inside crack initiates. Then, the inside crack grows both towards the contact surface and towards the center of the specimen. Finally, the surface crack unites with the inside crack at a depth of 0.10 mm at a low bulk load level (T3) or 0.13 mm at a high bulk load level (T6). Thus, a high bulk load seems to weaken the trend that the crack initiates inside the specimen under the high intensity of residual stress condition.

**Table 2:** Crack initiation angle for different computational cases.

Cases	T1	T2	T3	T4	T5	T6
**Crack Initiation Angle**	47°	49°	50°	46°	47°	48°

### 4.2. Fretting Fatigue Life

**Figure 7:**
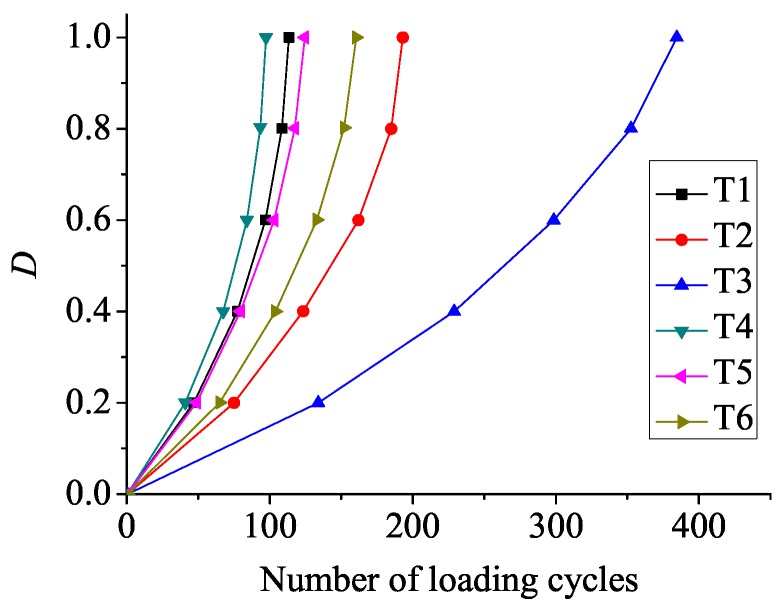
Damage (*D*) evolution in crack initiation sites located at the contact surface.

**Figure 8:**
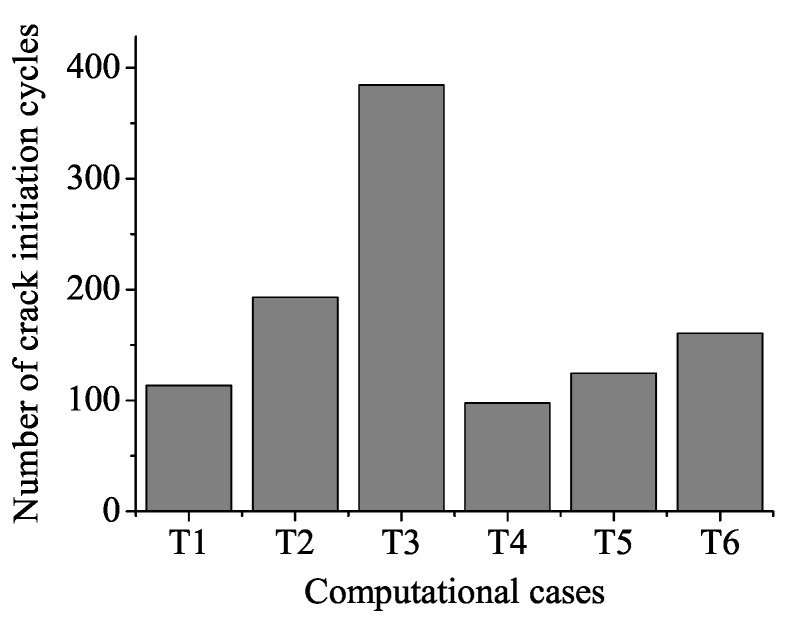
Fretting fatigue crack initiation life.

Material damage evolves gradually with the increase of loading cycles. Finally, the damage *D* reaches one, and the material fails. Figure 7 displays the damage evolution in crack initiation sites located at the contact surface. As Figure 7 suggested, residual compressive stress at contact surface diminishes material damage accumulation. Crack initiation life is the number of cycles when *D* = 1 at the crack initiation element on the contact surface. Loading cycles for crack initiation at the contact surface are 113.5, 193, 384.5, 97.5, 124.5, 160.5 from T1 to T6, respectively, as shown in Figure 8. The specimen in T4 endures a high bulk load and does not include residual stress, and therefore, damage accumulates the fastest. As a result, the specimen in T4 initiates prior to other specimens. Crack initiation life in specimens with a low and a high intensity of residual stress is enhanced by 70% (T2) and 238.8% (T3) compared to the specimen without residual stress (T1) at a low bulk load level, respectively. Crack initiation life in specimens with a low and a high intensity of residual stress is enhanced by 27.7% (T5) and 64.6% (T6) compared to the specimen without residual stress (T4) at a high bulk load level, respectively. In addition, under the fixed intensity of residual stress condition, crack initiation life is longer for specimens at a low bulk load level. Therefore, improvement of crack initiation life is more remarkable under the high intensity of residual stress condition and at a low bulk load level.

**Figure 9:**
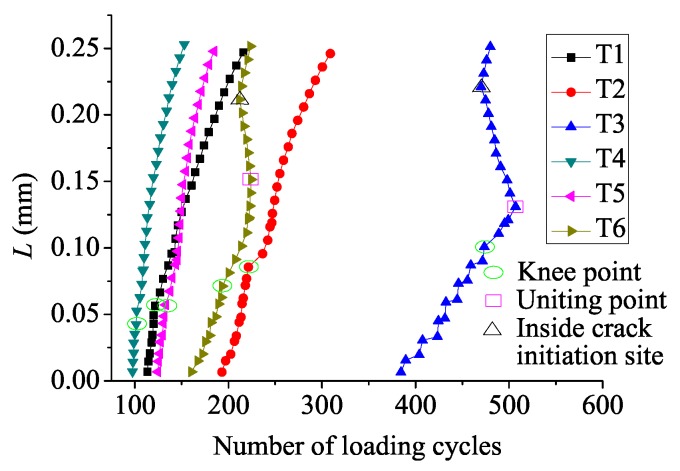
Fretting fatigue crack growth length (*L*) with the number of loading cycles.

Crack growth curves are depicted in Figure 9. Here, *L* stands for the length between the newly-formed crack tip and the specimen surface along the potential crack path. As Figure 9 suggested, for specimens without residual stress (T1 and T4) and specimens with a low intensity of residual stress (T2 and T5), curves are separated into two parts, *i.e.*, the curve before and after the knee point, which denote oblique and normal crack growth, respectively. For specimens with a high intensity of residual stress (T3 and T6), there are four segments, which are divided by the knee point, the uniting point and the inside crack initiation site.

As Figure 9 suggests, crack grows slower in the residual compressive stress zone, and the crack located in the residual tensile stress zone grows a little faster. However, the overall effects of residual stress enhance crack propagation life. When *L* reaches 0.25 mm, loading cycles for crack propagation are 102.5, 116, 122.5, 55.5, 60, 64 from T1 to T6, respectively. Crack propagation life in specimens with a low and a high intensity of residual stress (T2 and T3) is enhanced by 13.2% and 19.5% based on the specimen without residual stress (T1) at a low bulk load level, respectively. Crack propagation life in specimens with a low and a high intensity of residual stress (T5 and T6) is enhanced by 8.1% and 15.3% based on the specimen without residual stress (T4) at a high bulk load level, respectively. Total cycles in the whole loading process are 216, 309, 507, 153, 184.5, 224.5 from T1 to T6, respectively. Total life in specimens with a low and high intensity of residual stress (T2 and T3) is increased by 43.1% and 134.7% on the basis of the specimen without residual stress (T1) at a low bulk load level, respectively. Total life in specimens with a low and a high intensity of residual stress (T5 and T6) is increased by 20.6% and 46.7% on the basis of the specimen without residual stress (T4) at a high bulk load level, respectively. Therefore, a high intensity of residual stress has an advantage over a low intensity of residual stress in improving crack propagation life and total fretting fatigue life. In addition, crack propagation life and total life are longer for specimens at a low bulk load level under the fixed intensity of residual stress condition. In general, a high intensity of residual stress and a low bulk load lead to longer fretting fatigue life.

## 5. Conclusions

In this work, the effects of different intensities of residual stress on fretting fatigue at different bulk load levels are investigated. It is found that residual stress can affect the fretting fatigue crack growth path and increase fretting fatigue life. However, the increase of bulk load will offset the beneficial effects of residual stress on fretting fatigue life. Residual stress displays the most obvious effects for the case with a higher intensity of residual stress at a lower bulk load level, and in the studied case, crack initiation life is enhanced by 238.8% and total life by 134.7%. On the contrary, the case of a lower intensity of residual stress and a higher bulk load shows the worst improvement of fretting fatigue life. In this case, the crack initiation life and total life are enhanced only by 27.7% and 20.6%, respectively. This indicates that when the effects of residual stress on fretting fatigue life are estimated, the influences of bulk load also need to be considered.
